# Sequencing Depth Has a Stronger Effect than DNA Extraction on Soil Bacterial Richness Discovery

**DOI:** 10.3390/biom12030364

**Published:** 2022-02-25

**Authors:** Concepcion Sanchez-Cid, Romie Tignat-Perrier, Laure Franqueville, Laurence Delaurière, Trista Schagat, Timothy M. Vogel

**Affiliations:** 1 Environmental Microbial Genomics, Laboratoire Ampère, CNRS UMR 5005, Ecole Centrale de Lyon, Université de Lyon, 69134 Ecully, France; rom26.p@hotmail.fr (R.T.-P.); laure.franqueville@ec-lyon.fr (L.F.); vogel@univ-lyon1.fr (T.M.V.); 2 Scientific Applications and Training, Promega France, 69100 Charbonnières-les-Bains, France; laurence.delauriere@promega.com; 3 Institut des Géosciences de l’Environnement, Université Grenoble Alpes, CNRS, IRD, Grenoble INP, Grenoble, 38400 Saint-Martin-d’Hères, France; 4 Scientific Applications and Training, Promega Corporation, Madison, WI 53711, USA; trista.schagat@promega.com

**Keywords:** sequencing depth, DNA extraction, soil microbiome, bacterial richness, metagenomics

## Abstract

Although Next-Generation Sequencing techniques have increased our access to the soil microbiome, each step of soil metagenomics presents inherent biases that prevent the accurate definition of the soil microbiome and its ecosystem function. In this study, we compared the effects of DNA extraction and sequencing depth on bacterial richness discovery from two soil samples. Four DNA extraction methods were used, and sequencing duplicates were generated for each DNA sample. The V3–V4 region of the 16S rRNA gene was sequenced to determine the taxonomical richness measured by each method at the amplicon sequence variant (ASV) level. Both the overall functional richness and antibiotic resistance gene (ARG) richness were evaluated by metagenomics sequencing. Despite variable DNA extraction methods, sequencing depth had a greater influence on bacterial richness discovery at both the taxonomical and functional levels. Sequencing duplicates from the same sample provided access to different portions of bacterial richness, and this was related to differences in the sequencing depth. Thus, the sequencing depth introduced biases in the comparison of DNA extraction methods. An optimisation of the soil metagenomics workflow is needed in order to sequence at a sufficient and equal depth. This would improve the accuracy of metagenomic comparisons and soil microbiome profiles.

## 1. Introduction

The soil ecosystem arguably harbours the highest diversity of microorganisms of any ecosystem [1]. Unravelling the composition and function of the soil microbiome is critical to better understanding the role of these microbial communities in soil function and ecosystem services. The use of Next Generation-Sequencing (NGS) techniques has increased our access to the microbial communities present in soil, especially to the large proportion of uncultured microorganisms [2,3]. Nevertheless, each methodological step from soil sampling to sequence annotation presents inherent biases that limit the depth and reliability of soil microbiome analyses [4,5,6,7]. Of all these biases, the ones associated with DNA extraction have been particularly highlighted for their effects [8,9]. DNA can be adsorbed by soil compounds such as clay [10,11,12], which, when combined with the presence of lysis-recalcitrant bacteria [13], reduces DNA extraction efficiency. Moreover, organic matter and humic acids that are known to potentially inhibit enzymatic reactions [14,15] are often coextracted with DNA. Many studies have compared DNA extraction methods and documented the biases imposed by lysis procedures [16,17,18,19]. This has provoked proposals of different methods for DNA extraction and purification from soil over the past few decades [16,20,21,22] in an attempt to obtain an unbiased picture of soil microbiome biodiversity [10]. Nevertheless, no method has been shown to overcome all the biases described above, and the debate on the choice of DNA extraction methods is still ongoing.

The criteria used to define the performance of a DNA extraction method vary between studies and range from nucleic acid yield to phylogenetic diversity. However, higher yields, purity, and integrity do not always imply an improvement in bacterial diversity discovery [5]. Analyses of the relative abundance of taxonomic groups have a limited potential for selecting nucleic acid extraction methods, since the biases associated with soil metagenomics prevent us from determining the actual distribution of soil microbial populations within a community [23]. Furthermore, nucleic acid extraction methods may modify the relative abundance of detected communities without affecting the bacterial richness discovery [24]. In other words, the use of different DNA extraction methods might detect different proportions of the same communities [25] rather than different taxa or functions. The goal of this paper was not to resolve the debate concerning the use of relative abundance versus richness. We consider that bacterial richness measurements provide a more objective comparison of the performance of nucleic acid extraction methods, since biodiversity calculations with a limited set of sequences are strongly biased by evenness, which depends on the number of sequences. In addition, the actual relative abundances of the soil microbiome remain unknown.

The sequencing depth has also shown an important effect on bacterial discovery. Several studies have observed that low sequencing depths may bias the evaluation of the composition and function of microbial communities, both in terms of richness and diversity [26,27,28]. However, to the best of our knowledge, this is the first study to compare simultaneously the relative contribution of both sequencing depth and DNA extraction to soil bacterial richness discovery. DNA was extracted from two soil samples using four DNA extraction methods: two novel semiautomated methods for DNA extraction, a commercial kit, and the phenol/chloroform method as described by Griffiths et al. [29]. Sequencing duplicates were generated for each DNA sample to account for the effects of sequencing depth on bacterial richness discovery, which was evaluated at three levels. First, taxonomic richness obtained from the sequencing of the V3–V4 hypervariable region of the 16S rRNA gene was measured at the amplicon sequence variant (ASV) level. Second, the overall functional richness was determined from metagenomic samples annotated using the second level of classification of the SEED hierarchical subsystems [30]. Finally, the richness discovery of antibiotic resistance genes (ARGs) annotated from metagenomic reads using the CARD database [31] was also assessed. These genes are not abundant and are often associated with Mobile Genetic Elements (MGE) [32,33,34], which affects their identification using metagenomics and their association to bacterial hosts [35]. Given ARG’s relatively low abundance in the environment, the sequencing depth could have a stronger impact on their richness discovery than on the overall functional richness discovery.

## 2. Materials and Methods

### 2.1. Soil Sampling

Two soils were selected for this study (Table 1). Soils were sampled at an experimental farm (Scottish Agricultural College, Craibstone, Scotland, grid reference NJ872104) and at a field planted with corn at La Côte Saint Andre, France. All samples were kept at 4 °C before DNA extraction. Details about the Scottish Agricultural College soil composition were provided by Kemp et al. [36]. Physical characterisation of the soil from La Côte de Saint André was performed by CESAR (Centre Scientifique Agricole Régional) using the standard methods (NFX 31-107, NFX 31-117, ISO 10694, and ISO 13878). According to the national legislation in both Scotland and France, where the samples were collected, no ethical application was needed for the collection of soil samples.

### 2.2. DNA Extraction and Purification

DNA was extracted from 250 mg of sample using the DNeasy PowerSoil Kit (QIAGEN, Hilden, Germany) and the phenol/chloroform extraction method described by Griffiths et al. [29], as well as a new semiautomated protocol in which the Maxwell RSC Instrument (Promega, Madison, WI, USA) and a prototype version of the Maxwell RSC Fecal Microbiome DNA Kit (Promega) are used for DNA purification. Two modifications of this protocol were tested and are referred to as the Maxwell 1 and Maxwell 2 methods. All DNA extractions were performed in triplicate. In the Maxwell 1 method, 250 mg of sample were diluted in 1 mL of Lysis Buffer (Promega) and heated for 5 min at 95 °C. Samples underwent bead-beating twice at 5.5 m/s for 30 s in Lysis Matrix E tubes (MP Biomedicals, Irvine, CA, USA) and centrifuged at 10,600× *g* for 5 min. Then, 300 µL of supernatant were added to 300 µL of Binding Buffer (Promega) and loaded into a Maxwell RSC cartridge containing magnetic beads for DNA purification on the Maxwell RSC Instrument, according to Technical Manual TM640. A second purification using the ProNex Size-Selective Purification System (Promega) was carried out to reduce the humic acid carryover. In the Maxwell 2 method, two variants were introduced in the previously described protocol: 500 µL of Lysis Buffer were mixed with 500 µL of 0.5-M Sodium Phosphate Buffer (pH 7.0) and added to 250 mg of sample, and cells were lysed without bead-beating.

### 2.3. Quantitative PCR (qPCR) Assay

DNA was quantified using the Qubit Fluorometer and the Qubit dsDNA HS Assay Kits (Thermo Fisher Scientific, Waltham, MA, USA) and diluted to 2.5 ng/µL. Total bacterial abundance was estimated by quantifying the V3 region of the 16S rRNA gene by qPCR using the “universal” primers 341F (5′-CCT ACG GGA GGC AGC AG-3′) and 534R (5′-ATT ACC GCG GCT GCT GGC A-3′) [37,38]. A qPCR assay was carried out using the Corbett Rotor-Gene 6000 (QIAGEN) in a 20-µL reaction volume containing GoTaq qPCR Master Mix (Promega), 0.75 µM of each primer, and 2 µL of DNA at 2.5 ng/µL (5 ng of DNA). Two non-template controls were also included. The standard curve was obtained using 10-fold serial dilutions (10^7^ to 10^2^ copies) of a linearised plasmid pGEM-T Easy Vector (Promega) containing the 16S rRNA gene of *Pseudomonas aeruginosa* PAO1. Cycling conditions for qPCR amplification were 95 °C for 2 min, followed by 30 cycles of 95 °C for 15 s, 60 °C for 30 s, and 72 °C for 30 s. Melting curves were generated after amplification by increasing the temperature from 60 °C to 95 °C. 

### 2.4. 16S rRNA Gene Amplicon Sequencing and Analysis

All DNA samples were diluted to 2.5 ng/µL before 16S rRNA gene PCR amplification and sequencing to reduce PCR inhibition and normalise the amount of starting material for library preparation. Then, the V3–V4 hypervariable region of the bacterial 16S rRNA gene was amplified using Titanium Taq DNA Polymerase (Takara Clontech, Kyoto, Japan) and forward 341F with Illumina overhang (5′-TCG TCG GCA GCG TCA GAT GTG TAT AAG AGA CAG TCG TCG GCA GCG TCA GAT GTG TAT AAG AGA CAG CCT ACG GGN GGC WGC AG-3′) and reverse 785F with Illumina overhang (5′-GTC TCG TGG GCT CGG AGA TGT GTA TAA GAG ACA GGT CTC GTG GGC TCG GAG ATG TGT ATA AGA GAC AGG ACT ACH VGG GTA TCT AAT CC-3′) primers [6] to identify the Amplicon Sequence Variants (ASVs). Cycling conditions for PCR amplification were 95 °C for 3 min, followed by 25 cycles of 95 °C for 30 s, 55 °C for 30 s, and 72 °C for 30 s and a final extension step at 72 °C for 5 min. DNA libraries were prepared based on Illumina’s “16S Metagenomics Library Prep Guide” (15044223 Rev. B) using the Platinum Taq DNA Polymerase (Invitrogen, Waltham, MA, USA) and the Nextera XT Index Kit V2 (Illumina, San Diego, CA, USA). DNA sequencing with a 15% PhiX spike-in was performed using the MiSeq System and the MiSeq Reagent Kit v2 (Illumina). All samples were sequenced twice (in two different runs following the same protocol), except two samples extracted from La Côte de Saint André—one using the Maxwell 1 method and another one using the phenol/chloroform method—for which there was no DNA left for a second sequencing run. Sequences were trimmed, filtered, and denoised, ASVs were inferred, and chimeras were removed using DADA2 [39]. Since the amplicon size was too big to ensure the proper merging of forward and reverse reads, only forward reads were used in this analysis. Nineteen nucleotides and 10 nucleotides were trimmed from the left and right ends of the forward reads, respectively. Reads were filtered to have 0 Ns and a maximum of 2 expected errors. After following all the steps of the DADA2 pipeline, singletons were removed from inferred ASVs. In order to evaluate the effect of the sequencing depth in the taxonomic richness assessment, the ASV richness detected in each sample was determined using the vegan package (version 2.5–6) [40] in R (version 3.5.1) and plotted as a function of the sequencing depth. Finally, Venn diagrams comparing the taxonomic richness discovery of different DNA extraction methods, sequencing runs, individual samples sequenced at different depths, and samples extracted using different methods and sequenced at similar depths (a maximum difference of 1000 sequences between samples) were obtained using the VennDiagram package (version 1.6.20) [41] in R.

### 2.5. Metagenomics Sequencing and Analysis

Metagenomics libraries were prepared from <1 ng of DNA using the Nextera XT Library Prep Kit and Indexes (Illumina), as detailed in Illumina’s “Nextera XT DNA Library Prep Kit” reference guide (15031942 v03). DNA sequencing with a 1% PhiX spike-in was performed using the MiSeq System and the MiSeq Reagent Kit v2 (Illumina). All samples were sequenced twice (in two different runs following the same protocol), except two samples extracted from La Côte de Saint André—one using the Maxwell 1 method and another one using the phenol/chloroform method—for which there was no DNA left for a second sequencing run. Metagenomics reads were trimmed using the Fastq Quality Trimmer tool of the FASTX Toolkit. Nucleotides that did not meet a minimum quality score of Q20 were trimmed from the sequences, and sequences shorter than 100 nucleotides after trimming were removed. Two samples extracted using the DNeasy PowerSoil Kit—one from La Côte de Saint André and one from the Scottish Agricultural College—were removed from the analysis, since they did not meet a sequencing depth of 10,000 sequences. Reads were blasted against the nr database using Diamond [42] and filtered at an e-value of 10^−5^, an identity of 60%, and a query cover of 70%. The best hit was selected. Then, the sequences were functionally annotated using MEGAN6 [43] and the SEED hierarchical subsystems [30]. The second level of hierarchical functional subsystems classification was selected for functional class richness discovery analysis, and singletons were removed. In parallel, reads were blasted against the CARD database [31] using Diamond and filtered at an identity of 60%, a length of 33 amino acids, and an e-value of 10^−5^. The best hit was chosen, and singletons were removed. To evaluate the effect of the sequencing depth in both the overall function and ARG richness discovery, the functional class richness and the ARG richness detected in each sample were determined using the vegan package in R and plotted as a function of the sequencing depth. Finally, Venn diagrams comparing the taxonomic richness discovery of different DNA extraction methods, sequencing runs, individual samples sequenced at different depths, and samples extracted using different methods and sequenced at similar depths (a maximum difference of 1000 sequences between samples) were obtained using the VennDiagram package (version 1.6.20) in R.

### 2.6. Statistical Analysis

Correlations between sequencing depth and measured ASV and functional and ARG richness were determined using the Pearson coefficient, and the significance of the correlation was evaluated by a two-tailed Student’s *t*-distribution. Samples from each of the two soils were then grouped by a sequencing run or by DNA extraction method to determine the effect of these factors on ASV and functional and ARG richness discovery (Tables S1–S6 in the Supporting Information). Data normality was checked using the Shapiro–Wilk test. Statistical differences in richness between the conditions (sequencing run or DNA extraction method) were evaluated using ANOVA and pairwise two-tailed *t*-Student tests when data showed a normal distribution and using Kruskal–Wallis and pairwise Wilcoxon signed-rank tests when the data showed a non-normal distribution. Only significant differences are shown for pairwise comparisons.

## 3. Results

### 3.1. Total Bacterial Abundance Extracted by Each Method

The total abundance of the bacterial community extracted from two different soils, which was estimated by the number of copies of the 16S rRNA gene, varied between DNA extraction methods. Higher numbers of 16S rRNA gene copies were extracted from both soils using the phenol/chloroform method (Table 2). On the other hand, lower numbers of 16S rRNA gene copies were obtainedfrom the Scottish Agricultural College using the Maxwell 2 modification of the prototype Maxwell RSC Fecal Microbiome DNA Kit (Promega) and from La Côte de Saint André soil using the DNeasy PowerSoil Kit (QIAGEN) compared to using any other method on the same soil.

### 3.2. Sequencing Depth and DNA Extraction Effect on Bacterial Richness Discovery

**Figure 1:**
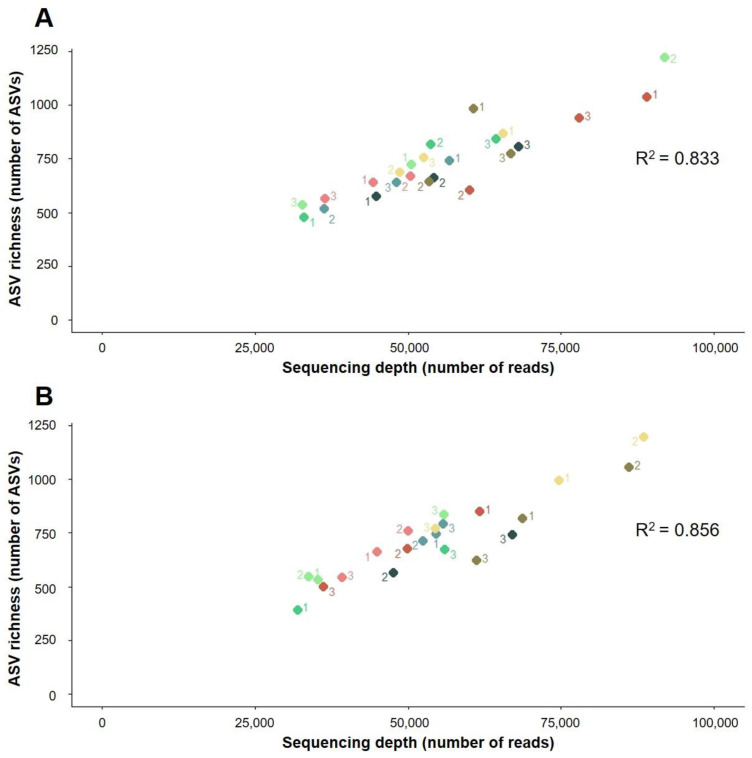
Effect of sequencing depth on the taxonomic richness discovery from (**A**) the Scottish Agricultural College soil (Craibstone, Scotland) and (**B**) La Côte de Saint André soil (France). Green: Maxwell 1 modification of the prototype Maxwell RSC Fecal Microbiome DNA Kit (Promega). Red: Maxwell 2 modification of the prototype Maxwell RSC Fecal Microbiome DNA Kit (Promega). Blue: Phenol/Chloroform. Yellow: DNeasy PowerSoil Kit (QIAGEN). The taxonomical richness measured in each sample was determined and plotted as a function of the sequencing depth. For each method/soil pair, triplicates (labelled 1, 2, and 3) were sequenced twice and plotted in the graph. Lighter colours represent the first sequencing run, and darker colours represent the second sequencing run. Correlation *p*-value: 5.15 × 10^−10^ (Scottish Agricultural College soil) and 7.38 × 10^−10^ (La Côte de Saint André soil).

**Figure 2:**
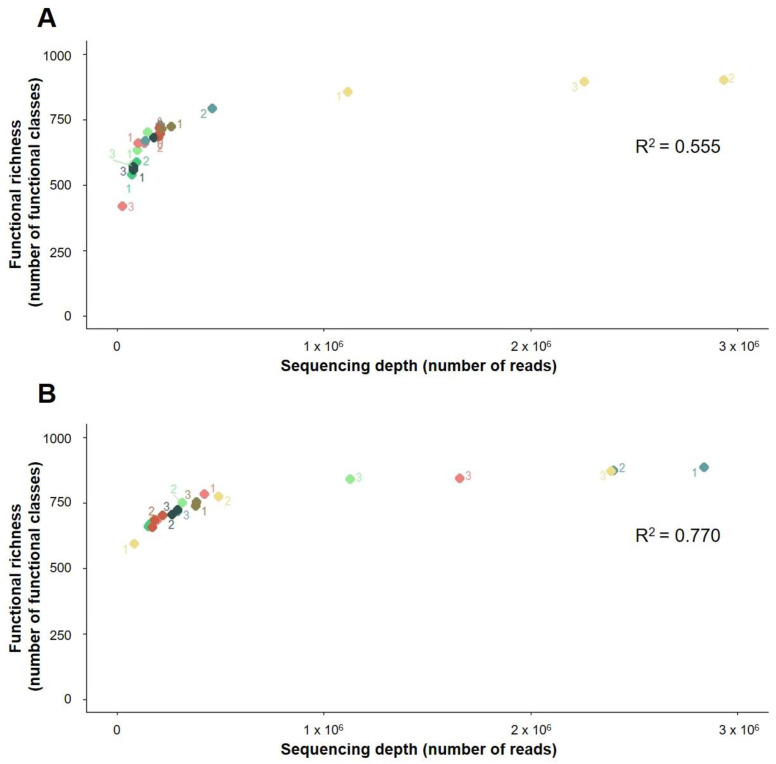
Effect of sequencing depth on the functional richness discovery from (**A**) the Scottish Agricultural College soil (Craibstone, Scotland) and (**B**) La Côte de Saint André soil (France). Green: Maxwell 1 modification of the prototype Maxwell RSC Fecal Microbiome DNA Kit (Promega). Red: Maxwell 2 modification of the prototype Maxwell RSC Fecal Microbiome DNA Kit (Promega). Blue: Phenol/Chloroform. Yellow: DNeasy PowerSoil Kit (QIAGEN). The functional richness (second SEED sublevel of classification) measured in each sample was determined and plotted as a function of the sequencing depth. For each method/soil pair, triplicates (labelled 1, 2, and 3) were sequenced twice and plotted in the graph. Lighter colours represent the first sequencing run, and darker colours represent the second sequencing run. Correlation *p*-value: 4.55 × 10^−5^ (Scottish Agricultural College soil) and 1.72 × 10^−7^ (La Côte de Saint André soil).

**Figure 3:**
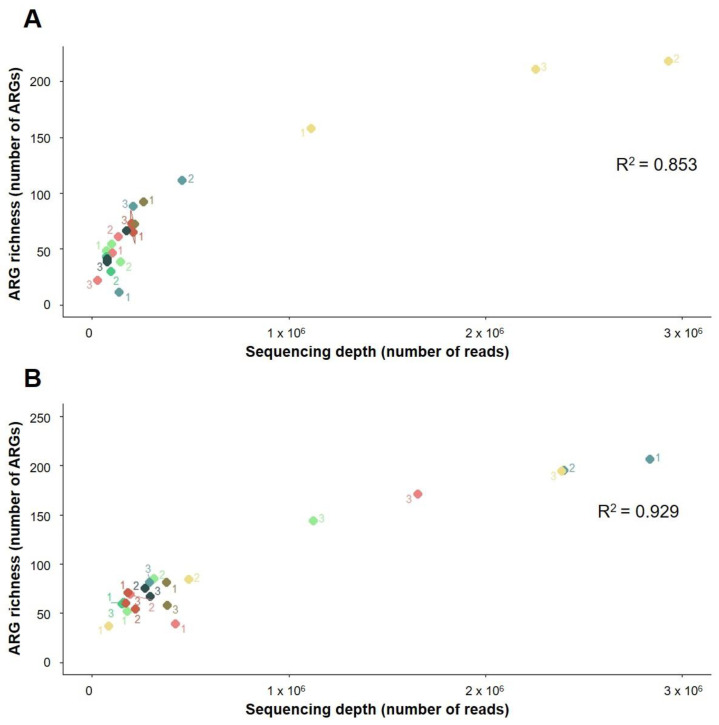
Effect of sequencing depth on the ARG richness discovery from (**A**) the Scottish Agricultural College soil (Craibstone, Scotland) and (**B**) La Côte de Saint André soil (France). Green: Maxwell 1 modification of the prototype Maxwell RSC Fecal Microbiome DNA Kit (Promega). Red: Maxwell 2 modification of the prototype Maxwell RSC Fecal Microbiome DNA Kit (Promega). Blue: Phenol/Chloroform. Yellow: DNeasy PowerSoil Kit (QIAGEN). The ARG richness measured in each sample was determined and plotted as a function of the sequencing depth. For each method/soil pair, triplicates (labelled 1, 2, and 3) were sequenced twice and plotted in the graph. Lighter colours represent the first sequencing run, and darker colours represent the second sequencing run. Correlation *p*-value: 3.37 × 10^−10^ (Scottish Agricultural College soil) and 2.35 × 10^−12^ (La Côte de Saint André soil).

The sequencing depth was significantly correlated to ASV and functional and ARG richness discovery in both soils (Figure 1, Figure 2 and Figure 3). A greater sequencing depth increased the taxonomic and functional richness discovery from the two soils included in this study, regardless of the method used for DNA extraction or the sequencing run (Figure 1, Figure 2 and Figure 3). In other words, at a cursory level, all methods detected a similar taxonomical, functional, and ARG richness at equal sequencing depths. Furthermore, DNA extraction triplicates that were sequenced at different depths had access to different proportions of bacterial DNA richness (Figure 1, Figure 2 and Figure 3), and similar results were observed when different depths were obtained between sequencing runs from individual samples (Figure 1, Figure 2 and Figure 3). On the other hand, at similar sequencing depths, slight differences in the bacterial richness discovery were sometimes found between samples, regardless of whether they were extracted using the same or different methods (Figure 1, Figure 2 and Figure 3). These differences were also found between sequencing duplicates from the same sample sequenced at similar depths: some examples of this are the ASV richness measured from samples 3 and 2 extracted from La Côte de Saint André soil using the Maxwell 1 and the Maxwell 2 modifications of the prototype Maxwell RSC Fecal Microbiome DNA Kit (Promega), respectively (Figure 1B).

The resequencing of the samples in two different runs did not have a significant impact on the ASV and ARG richness discovery, whereas significant differences were found between the functional richness measured from the La Côte de Saint André samples in the two sequencing runs (Tables S1–S3 in the Supporting Information). On the other hand, significant differences were observed in the ASV richness measured by some of the DNA extraction methods in La Côte de Saint André soil (Table S4 in the Supporting Information), as well as in functional and ARG richness in the Scottish Agricultural College soil (Tables S5 and S6 in the Supporting Information). Regarding the ASV richness discovery from La Côte de Saint André, significantly higher measurements were obtained from samples extracted using the DNeasy PowerSoil Kit (Table S4 in the Supporting Information), i.e., the most deeply sequenced method, than from the Maxwell 1 and 2 methods, which showed the lowest sequencing depths (Figure 1B). In addition, the metagenomic sequencing of samples extracted from the Scottish Agricultural College soil using the DNeasy PowerSoil Kit provided significantly higher functional richness than the Maxwell 1 method (Table S5 in the Supporting Information) and significantly higher ARG richness than both the Maxwell 1 and the Maxwell 2 methods (Table S6 in the Supporting Information). DNeasy PowerSoil Kit triplicates from the first metagenomic sequencing run showed a higher sequencing depth than any other sample, whereas all samples extracted using the Maxwell methods showed low sequencing depths (Figure 2A and Figure 3A). In the comparisons where the distribution of sequencing depths between DNA extraction methods was more even (ASV richness in the Scottish Agricultural College soil and functional and ARG richness in La Côte de Saint André soil), the DNA extraction methods did not show a significant effect on the richness discovery (Tables S4–S6 in the Supporting Information).

**Figure 4:**
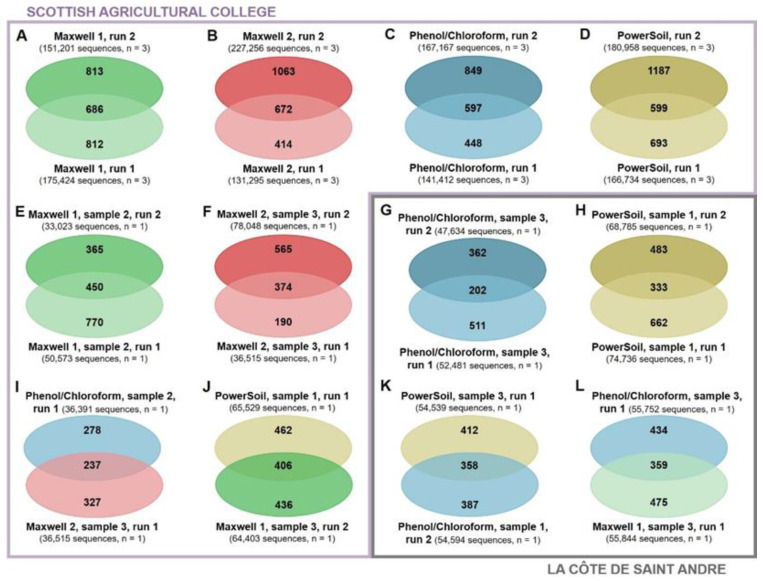
Venn Diagrams representing shared and unique ASVs from triplicates extracted using the same method sequenced in two different runs (**A**–**D**): individual samples sequenced at different depths (**E**–**H**) and samples extracted using different methods and sequenced at similar depths (**I**–**L**). Maxwell 1 and 2: modifications of the prototype Maxwell RSC Fecal Microbiome DNA Kit (Promega). PowerSoil: DNeasy PowerSoil Kit (QIAGEN).

**Figure 5:**
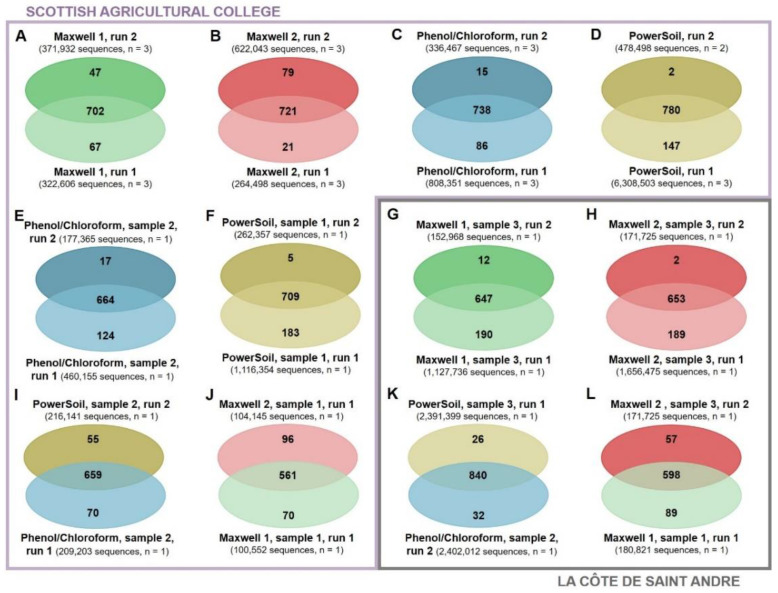
Venn Diagrams representing shared and unique functional classes from triplicates extracted using the same method sequenced in two different runs (**A**–**D**): individual samples sequenced at different depths (**E**–**H**) and samples extracted using different methods and sequenced at similar depths (**I**–**L**). Maxwell 1 and 2: modifications of the prototype Maxwell RSC Fecal Microbiome DNA Kit (Promega). PowerSoil: DNeasy PowerSoil Kit (QIAGEN).

**Figure 6:**
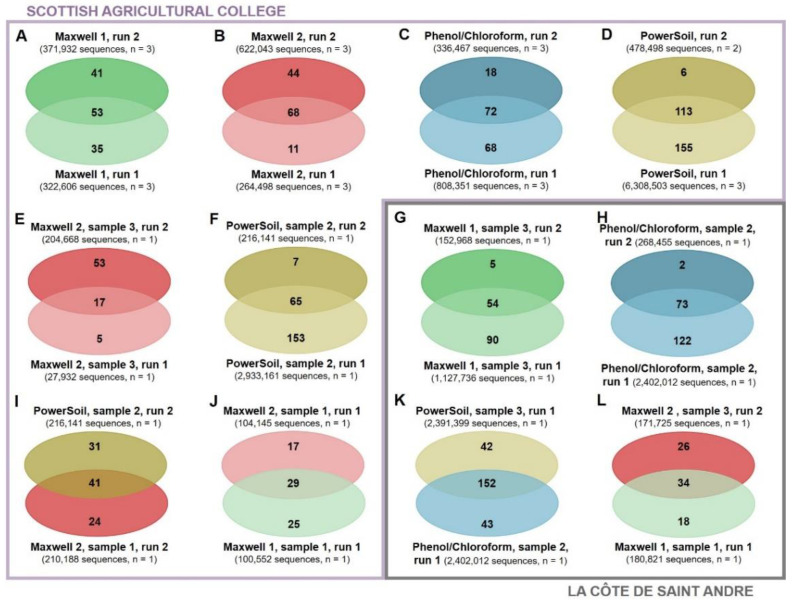
Venn Diagrams representing shared and unique ARGs from triplicates extracted using the same method sequenced in two different runs (**A**–**D**): individual samples sequenced at different depths (**E**–**H**) and samples extracted using different methods and sequenced at similar depths (**I**–**L**). Maxwell 1 and 2: modifications of the prototype Maxwell RSC Fecal Microbiome DNA Kit (Promega). PowerSoil: DNeasy PowerSoil Kit (QIAGEN).

The effects of sequencing depth and DNA extraction were compared at a more exhaustive level using Venn diagrams. At an overall level, the number of unique ASVs, functional classes and ARGs detected by a DNA extraction method increased with the sequencing depth (Figures S4–S6 in the Supporting Information). In addition, higher sequenced depths also increased the proportion of ASVs (Figure 4A–D), functional classes (Figure 5A–D), and ARGs (Figure 6A–D) discovered by the triplicates extracted using same method. This trend to detect more unique ASVs, functional classes, and ARGs at higher sequencing depths was observed for all the DNA extraction methods tested in this study. The sole exceptions to this were the similar ASV discoveries from triplicates extracted using the Maxwell 1 modification of the prototype Maxwell RSC Fecal Microbiome DNA Kit (Promega) and sequenced at different depths (Figure 4A) and the higher functional class discoveries obtained at lower sequencing depths from triplicates extracted using the same method (Figure 5A). When the same sample was sequenced twice and differences in the sequencing depth were observed, the richness discovery was also influenced. More unique ASVs (Figure 4E–H), functional classes (Figure 5E–H), and ARGs (Figure 6E–H) were identified when the sample was sequenced at a higher depth. These differences in richness discovery between the same sample increased with increasing differences in the sequencing depth and were more evident in the metagenomic sequencing, where the sequencing depth was more uneven (Figure 4E–H and Figure 5E–H). Finally, even though differences in the proportion of the soil ASV (Figure 4I–L) and overall functional (Figure 5I–L) and ARG (Figure 6I–L) richness unravelled by samples extracted using different methods and sequenced at similar depths were also found, the distribution of unique elements was more even than that observed when the same sample was sequenced at different depths. Regarding metagenomic sequencing, the number of functional classes and ARGs discovered by the two samples extracted using different methods and sequenced at a similar depth increased along with the sequencing depth (Figure 5I–L and Figure 6I–L). However, whereas the number of functional classes that were detected by only one sample decreased along with the sequencing depth (Figure 4I–L), at higher sequencing depths, both the number of core and unique discovered ARGs increased (Figure 6I–L).

## 4. Discussion

Although the development of soil metagenomic techniques implied a significant breakthrough in the understanding of soil microbial ecology, biases associated with these approaches prevent the accurate definition of the soil microbiome [44] and its associated functions in the soil ecosystem [45]. The aim of this study was to compare two main sources of biases in the metagenomic workflow: sequencing depth and DNA extraction methods and determine their relative contribution to bacterial richness discovery. Whereas sequencing depth had a significant impact on bacterial richness discovery from both soils analysed in the study at all the analysed levels, different sequencing runs and DNA extraction methods only showed significant differences when the sequencing depth was highly unequal between samples. In other words, these significant differences seem to be influenced by a higher sequencing depth of some samples rather than by the sequencing run or the DNA extraction method. Yet, clearly, when the sequencing depths were more evenly distributed between methods (i.e., when all methods showed a similar sequencing depth or all methods had at least one deeply sequenced sample), these differences became insignificant. This supports our hypothesis that the sequencing depth has a greater impact than the choice of the DNA extraction method on richness discovery at the ASV, functional class, and ARG level. 

The slight differences in the bacterial richness measured in different samples at similar sequencing depths could be partially due to DNA extraction biases if they were only observed between samples extracted using different methods. However, DNA extraction triplicates (Figure 1, Figure 2 and Figure 3) and sequencing duplicates (Figure 1B) sequenced at similar depths also detected different levels of bacterial richness. These differences could also be related to the distribution of ASVs, functional classes, and ARGs in soil ecosystems (Figures S1–S3 in the Supporting Information). Only a small proportion of annotated ASVs, functional classes, and ARGs are relatively abundant in the soils included in this study, while most of them are present at low abundances in extracted environmental DNA. This pattern of taxonomical distribution has already been observed in a study comparing soils from 237 different locations, where only 2% of the ensemble of bacterial phylotypes were found to be dominant [46]. Regarding functional classes, this distribution is consistent with a few functions being shared between different taxa and implicated in common bacterial ecology processes [47], while a large pool of low-abundant genes confers functions specific to single taxonomical groups. Finally, the few abundant ARGs in soil could have been selected, along with their carrying bacteria, in response to indigenous or exogenous selective pressures [48], whereas the majority of the natural reservoir of ARGs would represent a low-abundance pool. Since metagenomic techniques do not provide access to the whole bacterial diversity present in soil [49,50] and sequencing is a random subsampling from pools containing unevenly distributed taxa and genes, the proportion of high- and low-abundant elements sequenced from each sample could vary between samples, regardless of DNA extraction. This would lead to biased observations of bacterial richness that are likely more related to an insufficient sequencing depth than to DNA extraction limitations (Figures S4–S6 in the Supplementary Information).

The improved bacterial richness discovery from method triplicates (Figure 4A–D, Figure 5A–D and Figure 6A–D) and from individual samples (Figure 4E–H, Figure 5E–H and Figure 6E–H) at higher sequencing depths is consistent with a better detection of low-abundant elements at increasing sequencing depths. However, two exceptions were observed with this trend (Figure 4A and Figure 5A). These inconsistencies are an illustration of the random subsampling of pools leading to variations in the richness discovery. In addition, the more even distribution of unique elements between samples extracted using different methods and sequenced at similar depths (Figure 4I–L, Figure 5I–L and Figure 6I–L) than between sequencing duplicates from the same sample at different sequencing depths (Figure 4E–H, Figure 5E–H and Figure 6E–H) supports the idea that the sequencing depth has a stronger effect on the richness discovery than DNA extraction. Finally, whereas more core and less unique functional classes were detected by two extraction methods sequenced at equal depths when this depth increased (Figure 5I–L), and the number of both core and unique ARGs detected from both soils increased with the sequencing depth (Figure 6I–L). This supports the concern that short-read sequencing such as the one performed in this study provides an incomplete picture of the diversity of soil ARGs even at high sequencing depths and that the combination of long and short reads should be used to improve the ARG richness discovery [51,52].

Overall, our results confirm that the sequencing depth is a major source of biases in metagenomic studies and suggest that it has a stronger impact than DNA extraction on the richness discovery. Previous studies have shown that the sequencing depth has an impact on the bacterial richness and diversity discovery [26,27,28,53], but unfortunately, they were not coupled to the extraction method variants, since these have also been considered as critical for bacterial richness and diversity [16,17,18,54]. Thus, this is the first study (to the best of our knowledge) evaluating both effects simultaneously and demonstrating that the impact of sequencing depth on the soil bacterial richness discovery is greater than that of DNA extraction methods. This comparison can help determine which investments are more urgently needed to improve the metagenomics workflow. Whereas DNA extraction methods may affect the richness discovery, other factors, such as DNA amplification [55], library preparation, sequencing techniques [8], and sequence annotation [56], may also generate biases and lead to inaccurate comparisons between methods. We are currently unable to determine which of the differences observed between the DNA extraction methods are related to DNA extraction technique variability and which are a consequence of insufficient sequencing depths. Therefore, efforts should be made to optimise each of the steps in the metagenomic workflow in order to sequence representative samples of extracted DNA at a sufficient and even depth. This would not only facilitate an accurate comparison between DNA extraction methods but would also help define standard methods for soil metagenomics that would improve metagenomic comparisons and eventually lead to accurate profiles of soil microbiomes.

## Figures and Tables

**Table 1:** Physical characterisation of the samples selected for this study.

	Scottish Agricultural College	La Côte de Saint André
Sand	73.85%	42.9%
Silt	20.04%	43.6%
Clay	6.11%	13.5%
pH	4.5	7.24
Organic matter	5.97%	2.92%
Organic C	3.79%	1.7%
Total N	0.45%	0.17%

**Table 2:** 16S rRNA gene copy numbers from the Scottish Agricultural College (Craibstone, Scotland) and La Côte de Saint André (France) soils (250 mg) using different DNA extraction and purification strategies. Maxwell 1 and 2: modifications of the prototype Maxwell RSC Fecal Microbiome DNA Kit (Promega). All data (averages and standard deviations) are based on three separate soil samples for each method.

Soil	Method	16S rRNA Gene Copies/µL
Scottish Agricultural College soil	Maxwell 1	153.96 ± 52.41
	Maxwell 2	64.28 ± 9.13
	Phenol/Chloroform method	189.21 ± 15.04
	DNeasy PowerSoil Kit	165.31 ± 65.89
La Côte de Saint André soil	Maxwell 1	160.61 ± 14.7
	Maxwell 2	170.39 ± 16.32
	Phenol/Chloroform method	211.95 ± 45.41
	DNeasy PowerSoil Kit	88.9 ± 25.64

## Data Availability

The datasets generated and analysed during the current study are publicly available in the University of Lyon Environmental Microbial Genomics Group’s repository: https://www.mmnt.net/db/0/0/ftp.ec-lyon.fr/pub/ADN/. Last Accessed date 10 January 2022.

## Supplementary Materials

The following supporting information can be downloaded at https://www.mdpi.com/article/10.3390/biom12030364/s1: Figure S1: Abundance of the ensemble of ASVs from DNA extracted from (A) the Scottish Agricultural College soil and (B) La Côte de Saint André soil. Figure S2: Total abundance of the ensemble of functional classes classified using SEED from DNA extracted from (A) the Scottish Agricultural College soil and (B) La Côte de Saint André soil. Figure S3: Total abundance of the ensemble of ARGs from DNA extracted from (A) the Scottish Agricultural College soil and (B) La Côte de Saint André soil. Figure S4: Venn Diagrams representing shared and unique ASV from (A) the Scottish Agricultural College soil, both sequencing runs, (B) La Côte de Saint André soil, both sequencing runs, (C) the Scottish Agricultural College soil, first sequencing run, and (D) La Côte de Saint André soil, first sequencing run. Figure S5: Venn Diagrams representing shared and unique functional classes from (A) the Scottish Agricultural College soil, both sequencing runs, (B) La Côte de Saint André soil, both sequencing runs, (C) the Scottish Agricultural College soil, first sequencing run, and (D) La Côte de Saint André soil, first sequencing run. Figure S6: Venn Diagrams representing shared and unique ARGs from (A) the Scottish Agricultural College soil, both sequencing runs, (B) La Côte de Saint André soil, both sequencing runs, (C) the Scottish Agricultural College soil, first sequencing run, and (D) La Côte de Saint André soil, first sequencing run. Table S1: Statistics of the comparison between sequencing run effects on ASV richness discovery. Table S2: Statistics of the comparison between sequencing run effects on functional richness discovery. Table S3: Statistics of the comparison between sequencing run effects on ARG richness discovery. Table S4: Statistics of the comparison between DNA extraction method effects on ASV richness discovery. Table S5: Statistics of the comparison between DNA extraction method effects on functional richness discovery. Table S6: Statistics of the comparison between DNA extraction method effects on ARG richness discovery.
