# Supplementary material for: Sequencing Depth Has a Stronger Effect than DNA Extraction on Soil Bacterial Richness Discovery
Source: Biomolecules. 2022 Feb 25;12(3):364. doi: 10.3390/biom12030364 (PMC8945597; doi:10.3390/biom12030364)
Supplement: Supplementary file 1 [file biomolecules-12-00364-s001.zip › biomolecules-1572692-supplementary.pdf]

Supporting information

	All samples		Craibstone		St André	
	Run 1	Run 2	Run 1	Run 2	Run 1	Run 2
n	24	22	12	12	12	10
**Average richness**	735	729	713	763	757	688
**SD richness**	188	181	188	174	194	190
**SW test (*p*-value)**	0.08		0.23		0.52	
**T-test (*p*-value)**	0.92		0.51		0.41	

Table S1. Statistics of the comparison between sequencing runs effect on ASV richness discovery. Craibstone: Scottish Agricultural College soil (Craibstone, Scotland). St André: La Côte de Saint André soil (France). SD: standard deviation. SW: Shapiro-Wilk normality test. T-test: two-tailed Student's t-test.

	All samples		Craibstone		St André	
	Run 1	Run 2	Run 1	Run 2	Run 1	Run 2
n	24	20	12	11	12	9
**Average richness**	741	673	707	651	775	699
**SD richness**	121	62	140	72	92	35
**SW test (*p*-value)**	0.1449		0.41		0.27	
**T-test (*p*-value)**	**0.021**		0.24		**0.019**	

Table S2. Statistics of the comparison between sequencing runs effect on functional richness discovery. Craibstone: Scottish Agricultural College soil (Craibstone, Scotland). St André: La Côte de Saint André soil (France).SD: standard deviation. SW: Shapiro-Wilk normality test. T-test: two-tailed Student's t-test.

	All samples		Craibstone		St André	
	Run 1	Run 2	Run 1	Run 2	Run 1	Run 2
n	24	20	12	11	12	9
**Average richness**	101	62	89	60	113	65
**SD richness**	68	15	71	19	65	9
**SW test (*p*-value)**	5.67^10-6		0.0004		0.0003	
**W test (*p*-value)**	0.18		0.76		0.1	

Table S3. Statistics of the comparison between sequencing runs effect on ARG richness discovery. Craibstone: Scottish Agricultural College soil (Craibstone, Scotland). St André: La Côte de Saint André soil (France). SD: standard deviation. SW: Shapiro-Wilk normality test. W test: Wilcoxon signed-rank test.

	Craibstone				St André			
	Maxwell1	Maxwell2	Phenol/Chlo	PowerSoil	Maxwell1	Maxwell2	Phenol/Chlo	PowerSoil
n	6	6	6	6	5	6	5	6
**Av richness**	768	742	656	785	594	663	711	909
**SD richness**	266	197	106	124	167	131	87	211
**SW test (*p*-value)**	0.23				0.52			
**ANOVA (*p*-value)**	0.63				**0.021***			

Table S4. Statistics of the comparison between DNA extraction methods effect on ASV richness discovery. Craibstone: Scottish Agricultural College soil (Craibstone, Scotland). St André: La Côte de Saint André soil (France). Av: average. SD: standard deviation. SW: Shapiro-Wilk normality test. *The methods that showed significant differences in ASV richness discovery in La Côte de Saint André soil were the Maxwell 1 and the DNeasy PowerSoil Kit(*p*-value = 0.022, two-tailed Student's t-test) and the Maxwell 2 and the DNeasy PowerSoil Kit (*p*-value = 0.041, two-tailed Student's t-test).

	Craibstone				St André			
	Maxwell1	Maxwell2	Phenol/Chlo	PowerSoil	Maxwell1	Maxwell2	Phenol/Chlo	PowerSoil
n	6	6	6	5	5	6	5	5
**Av richness**	619	641	666	818	720	726	781	746
**SD richness**	64	111	91	92	75	73	90	100
**SW test (*p*-value)**	0.41				0.27			
**ANOVA (*p*-value)**	**0.0091***				0.66			

Table S5. Statistics of the comparison between DNA extraction methods effect on functional richness discovery. Craibstone: Scottish Agricultural College soil (Craibstone, Scotland). St André: La Côte de Saint André soil (France). Av: average. SD: standard deviation. SW: Shapiro-Wilk normality test. *The methods that showed significant differences in ASV richness discovery in La Côte de Saint André soil were the Maxwell 1 and the DNeasy PowerSoil Kit(*p*-value = 0.029, two-tailed Student's t-test).

	Craibstone				St André			
	Maxwell1	Maxwell2	Phenol/Chlo	PowerSoil	Maxwell1	Maxwell2	Phenol/Chlo	PowerSoil
n	6	6	6	5	5	6	5	5
**Av richness**	48	56	59	150	80	77	125	91
**SD richness**	15	19	37	67	38	47	69	61
**SW test (*p*-value)**	0.0004				0.0003			
**K test (*p*-value)**	**0.021***				0.37			

Table S6. Statistics of the comparison between DNA extraction methods effect on ARG richness discovery. Craibstone: Scottish Agricultural College soil (Craibstone, Scotland). St André: La Côte de Saint André soil (France). Av: average. SD: standard deviation. SW: Shapiro-Wilk normality test. *The methods that showed significant differences in ASV richness discovery in La Côte de Saint André soil were the Maxwell 1 and the DNeasy PowerSoil Kit(*p*-value = 0.0043, Wilcoxon signed-rank test) and the Maxwell 2 and the DNeasy PowerSoil Kit (*p*-value = 0.026, Wilcoxon signed-rank test).

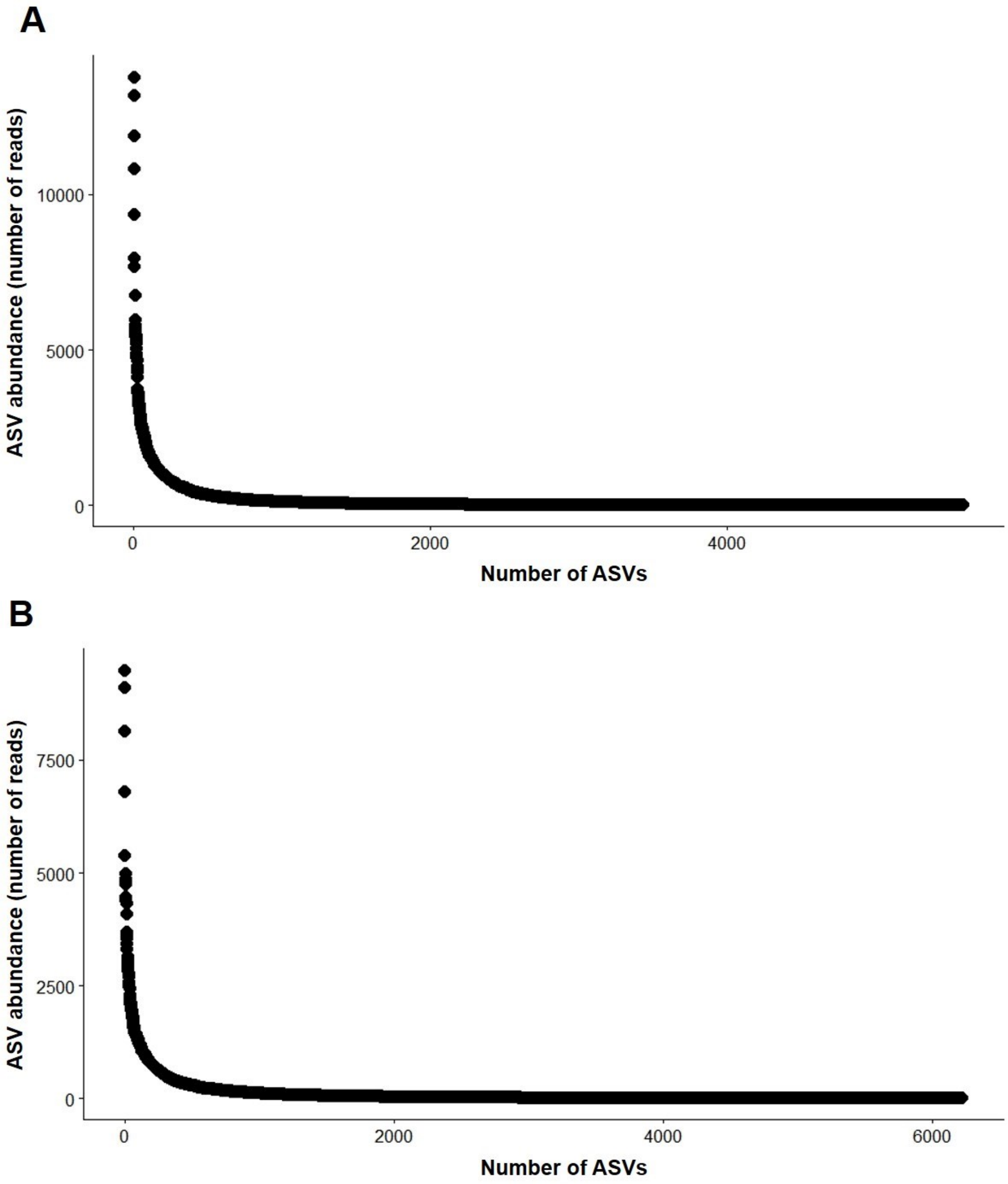

Figure S1. Total abundance of the ensemble of ASVs from DNA extracted from (A) the Scottish Agricultural College soil (Craibstone, Scotland); (B) La Côte de Saint André soil (France). The abundance of each measured ASV was determined and plotted in a decreasing order using R.

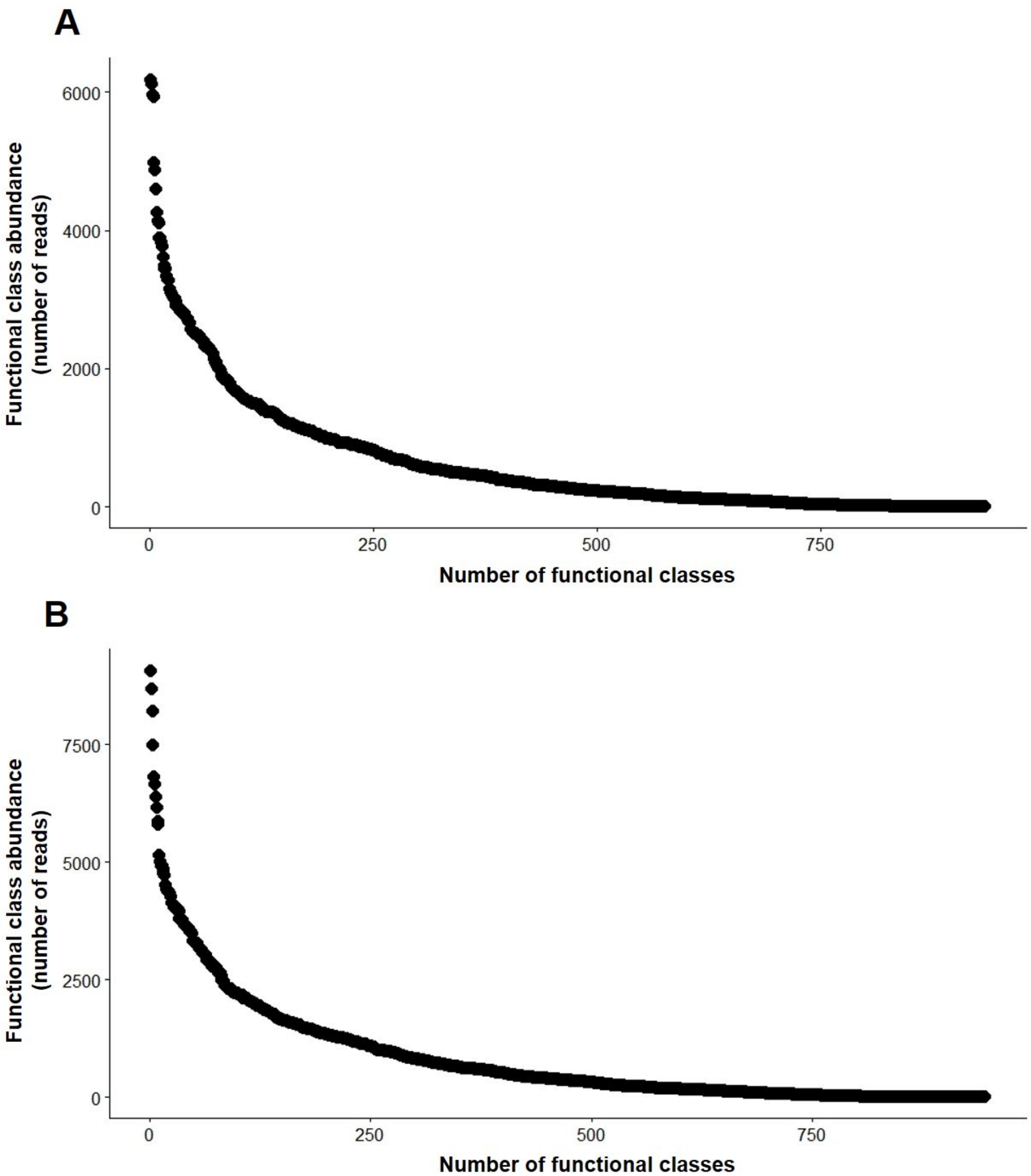

Figure S2. Total abundance of the ensemble of functional classes classified using SEED from DNA extracted from (A) the Scottish Agricultural College soil (Craibstone, Scotland); (B) La Côte de Saint André soil (France). The abundance of each measured functional class was determined and plotted in a decreasing order using R.

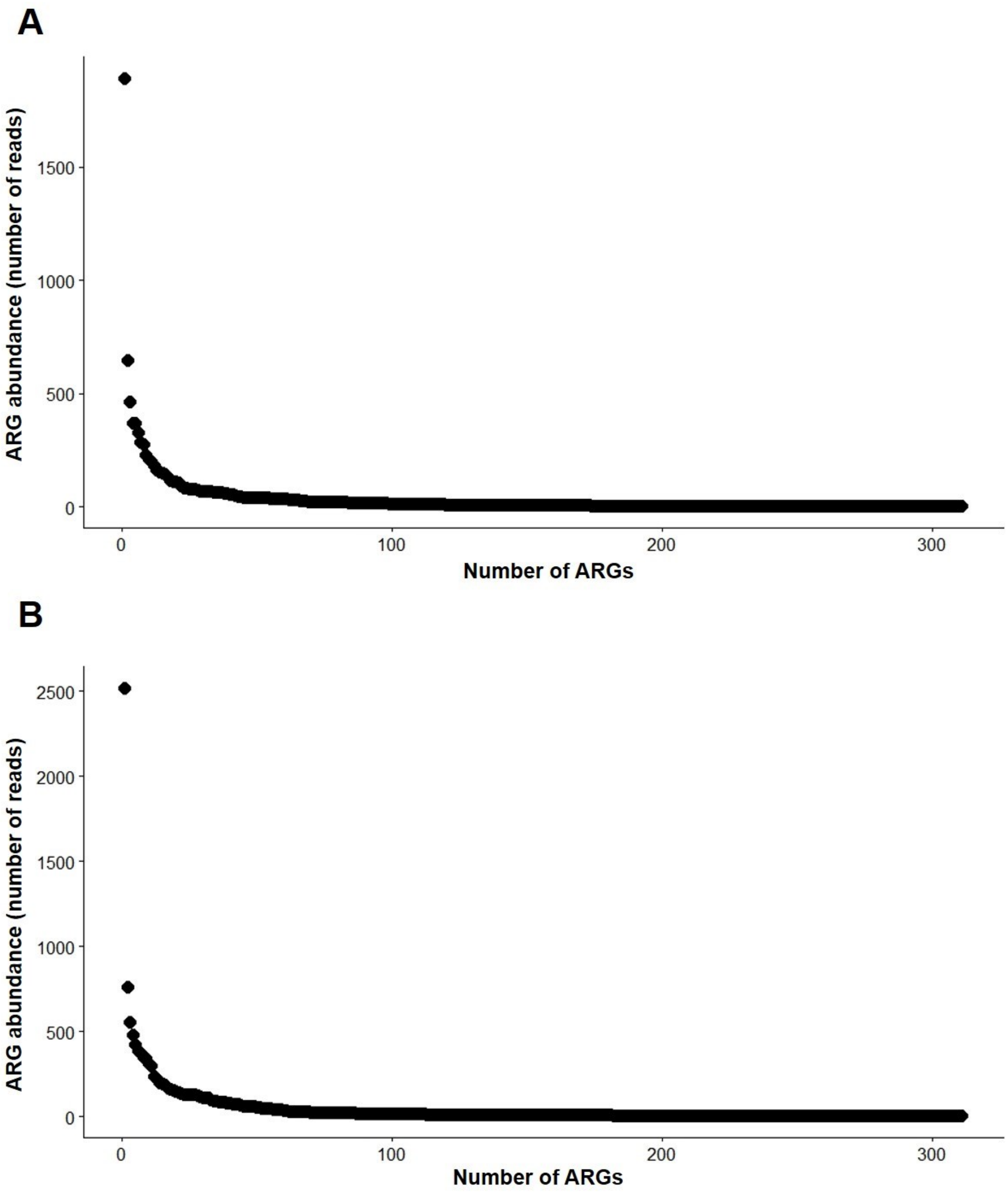

Figure S3. Total abundance of the ensemble of ARGs from DNA extracted from (A) the Scottish Agricultural College soil (Craibstone, Scotland); (B) La Côte de Saint André soil (France). The abundance of each measured ARG was determined and plotted in a decreasing order using R.

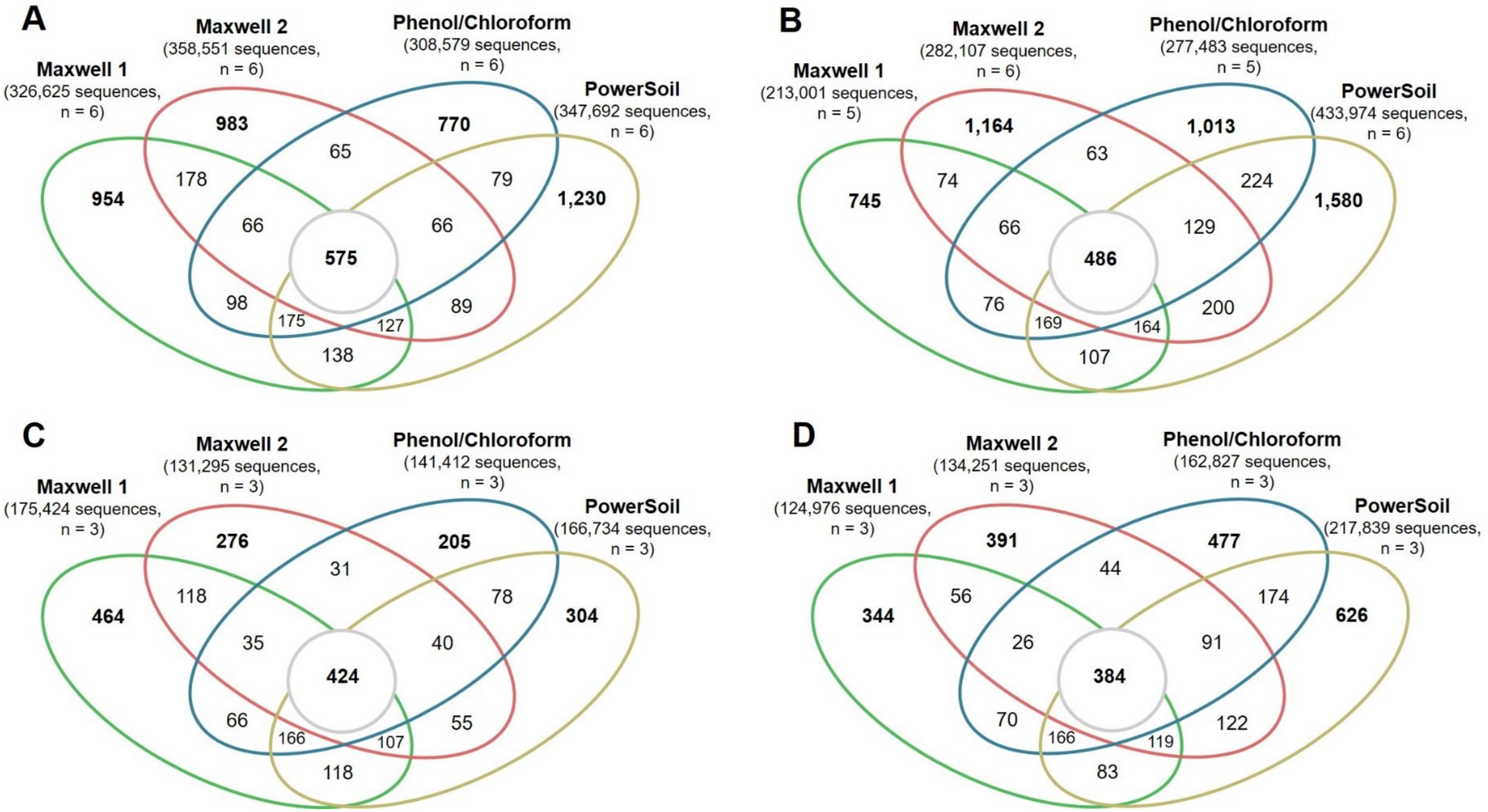

Figure S4. Venn Diagrams representing shared and unique ASV between methods for DNA extracted from (A) the Scottish Agricultural College soil (Craibstone, Scotland), both sequencing runs; (B) La Côte de Saint André soil (France), both sequencing runs; (C) the Scottish Agricultural College soil, first sequencing run; (D) La Côte de Saint André soil, first sequencing run. Maxwell 1 and 2: modifications of the prototype Maxwell RSC Fecal Microbiome DNA Kit (Promega). PowerSoilL: DNeasy PowerSoil Kit (QIAGEN).

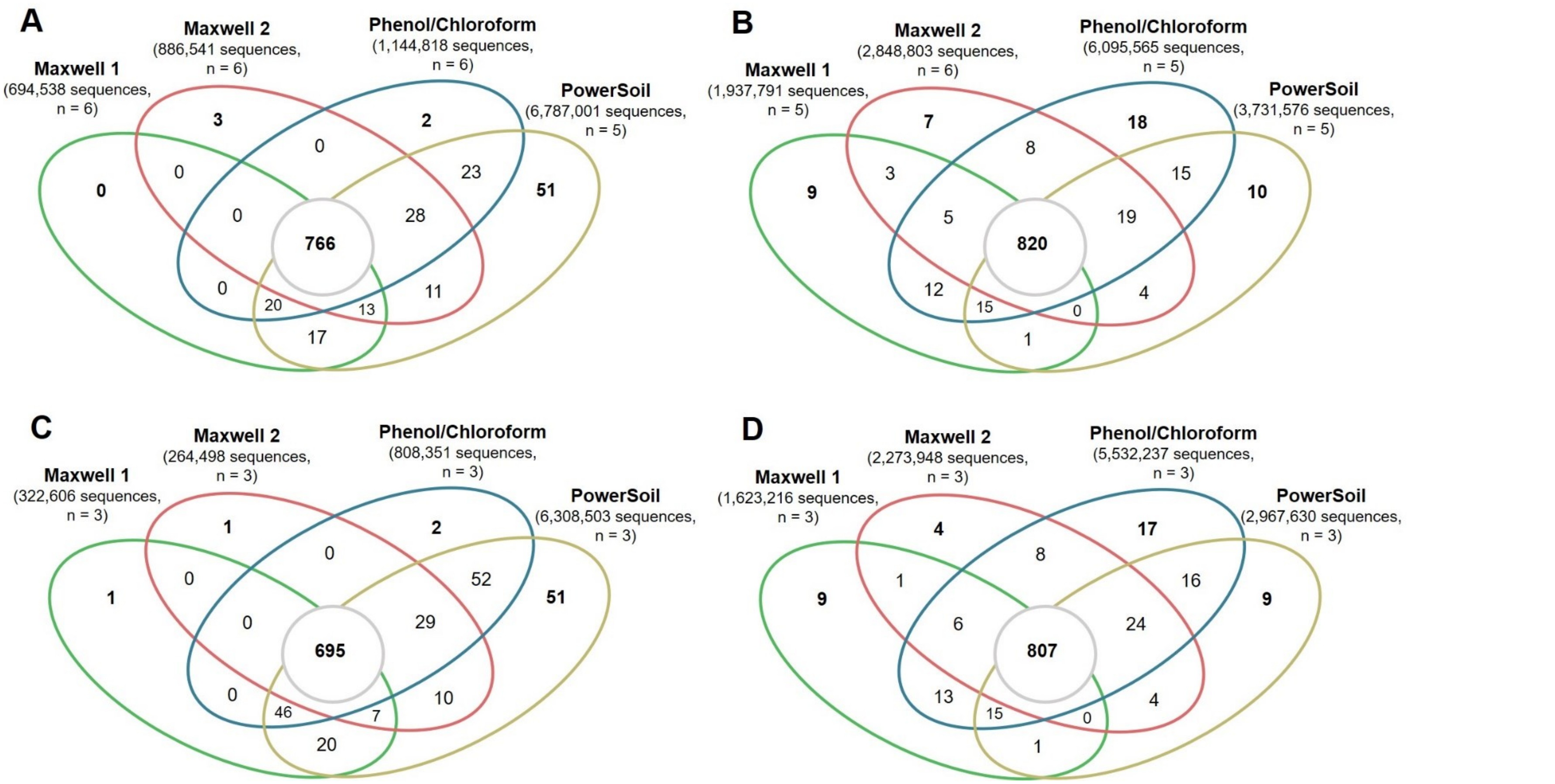

Figure S5. Venn Diagrams representing shared and unique functional classes between methods for DNA extracted from (A) the Scottish Agricultural College soil (Craibstone, Scotland), both sequencing runs; (B) La Côte de Saint André soil (France), both sequencing runs; (C) the Scottish Agricultural College soil, first sequencing run; (D) La Côte de Saint André soil, first sequencing run. Maxwell 1 and 2: modifications of the prototype Maxwell Fecal Microbiome Kit (Promega). PowerSoilL: DNeasy PowerSoil Kit (QIAGEN).

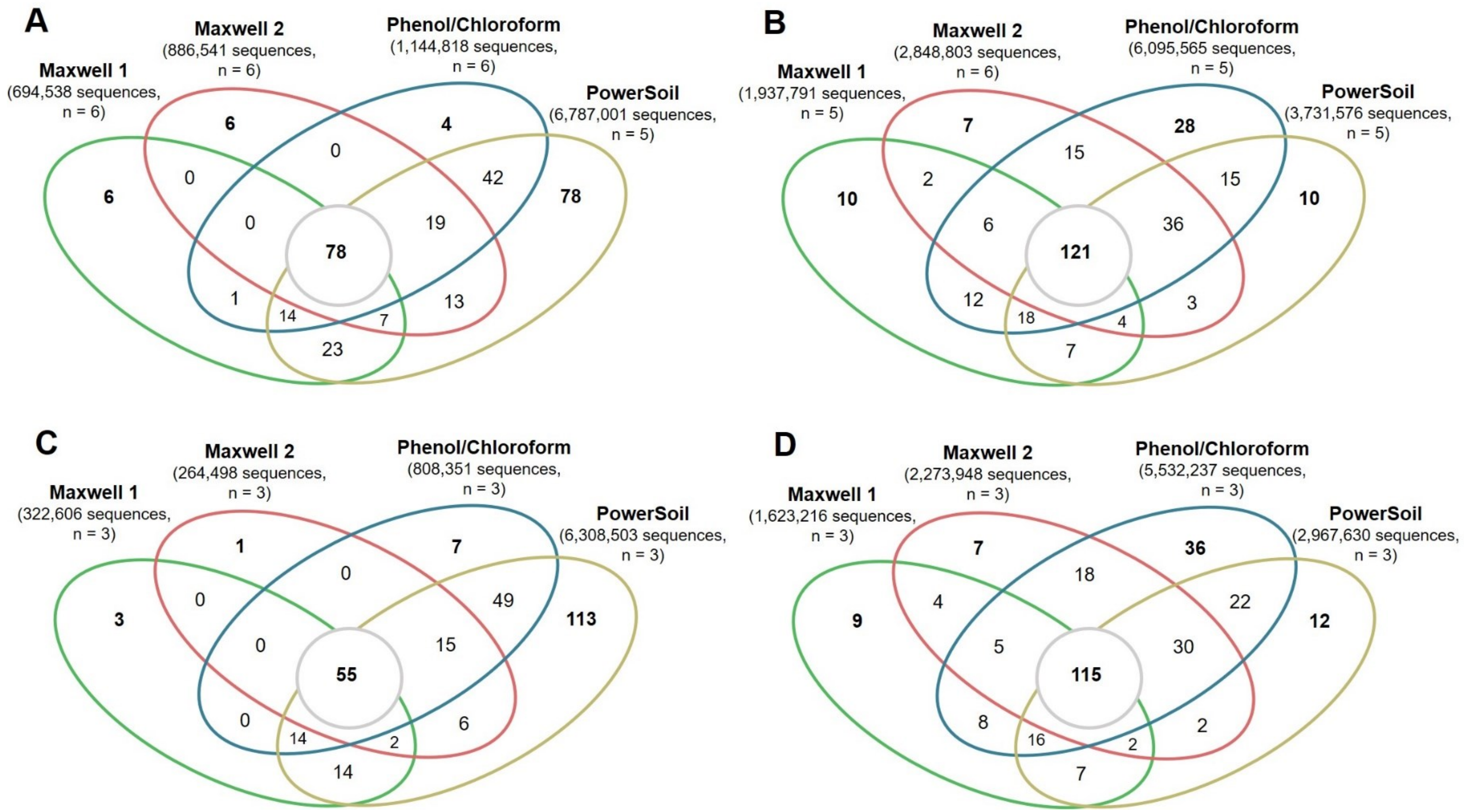

Figure S6. Venn Diagrams representing shared and unique ARGs between methods for DNA extracted from (A) the Scottish Agricultural College soil (Craibstone, Scotland), both sequencing runs; (B) La Côte de Saint André soil (France), both sequencing runs; (C) the Scottish Agricultural College soil, first sequencing run; (D) La Côte de Saint André soil, first sequencing run. Maxwell 1 and 2: modifications of the prototype Maxwell Fecal Microbiome Kit (Promega). PowerSoilL: DNeasy PowerSoil Kit (QIAGEN).
